# Supplementary material for: Saccharomyces boulardii (CNCM I-745) improves intestinal damage in sepsis by remodeling intestinal flora structure
Source: Front Cell Infect Microbiol. 2025 Jul 31;15:1602792. doi: 10.3389/fcimb.2025.1602792 (PMC12350329; doi:10.3389/fcimb.2025.1602792)
Supplement: Supplementary file 1 [file DataSheet1.zip › summary/summary/8_Advanced_analyse/2_PICRUSt2_result/COG/Group.DW4_vs_DW0_vs_CW4_vs_CW0_vs_BW4_vs_BW0_vs_AW4_vs_AW0/PICRUSt2_COG_Group.DW4_vs_DW0_vs_CW4_vs_CW0_vs_BW4_vs_BW0_vs_AW4_vs_AW0_diff.pdf]

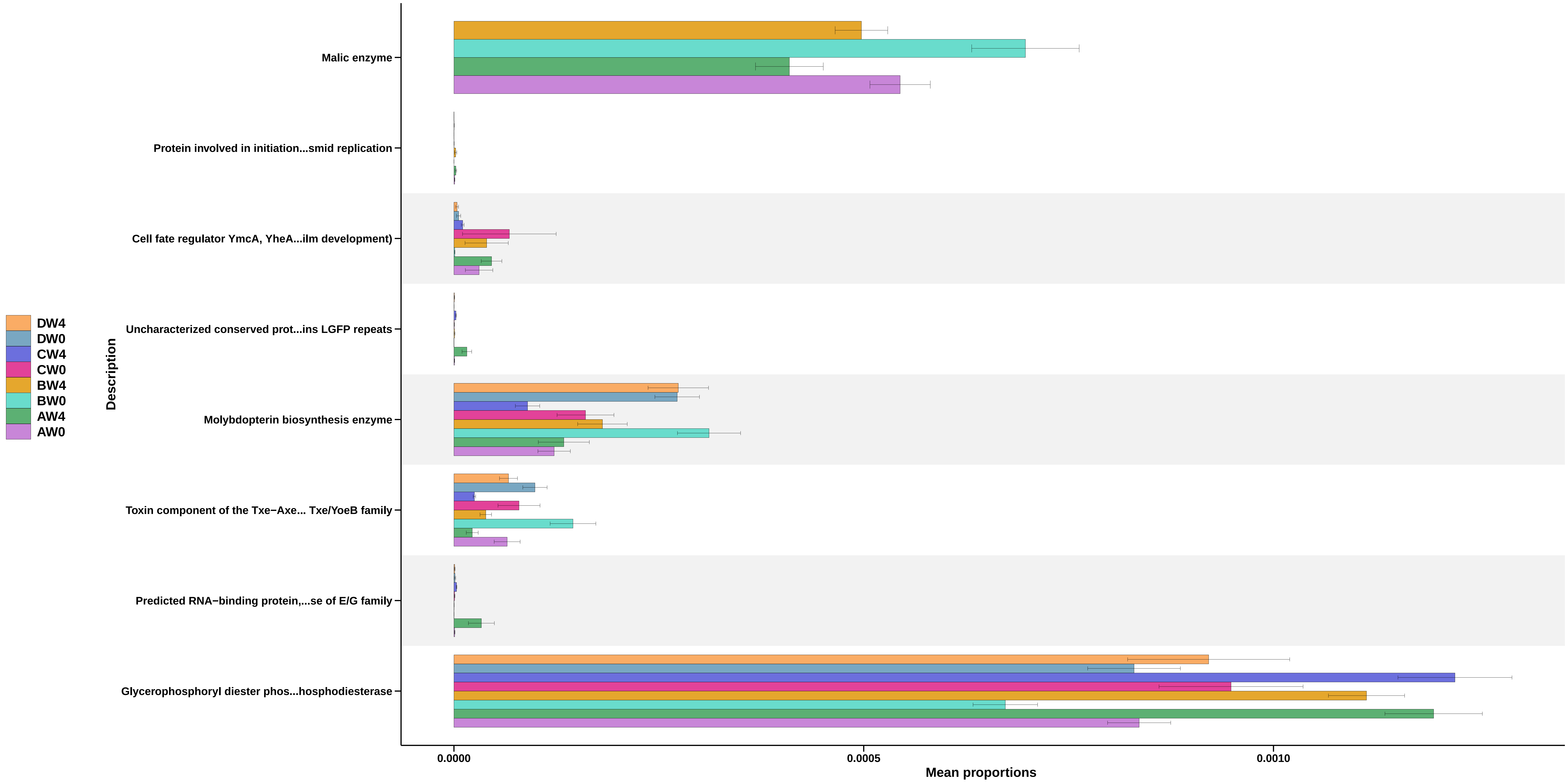

DW4
DW0
CW4
CW0
BW4
BW0
AW4
AW0
Description
Malic enzyme
Protein involved in initiation...smid replication
Cell fate regulator YmcA, YheA...ilm development)
Uncharacterized conserved prot...ins LGFP repeats
Molybdopterin biosynthesis enzyme
Toxin component of the Txe−Axe... Txe/YoeB family
Predicted RNA−binding protein,...se of E/G family
Glycerophosphoryl diester phos...hosphodiesterase
0.0000
0.0005
0.0010
Mean proportions
